# Antitumor Activity of Antimicrobial Peptides Containing C*iso*DGRC in CD13 Negative Breast Cancer Cells

**DOI:** 10.1371/journal.pone.0053491

**Published:** 2013-01-11

**Authors:** Lei Hou, Xinhan Zhao, Pei Wang, Qian Ning, Min Meng, Caigang Liu

**Affiliations:** 1 Department of Oncology, The First Affiliated Hospital of Medical School of Xi'an Jiaotong University, Xi'an, Shaanxi Province, China; 2 Department of Breast Surgery, General Surgery, the First Hospital of China Medical University, Shenyang, Liaoning Province, China; Wayne State University School of Medicine, United States of America

## Abstract

**Backgroud:**

*iso*Asp-Gly-Arg (*iso*DGR) is a derivative of the Asn-Gly-Arg (NGR) motif, which is used as a targeted delivery tool to aminopeptidase N (CD13) positive cells. Recent studies have shown that cyclic *iso*DGR (C*iso*DGRC) has a more efficient affinity with α_v_β_3_, a type of integrin that overexpresses in tumor cells. Antimicrobial peptides (AMPs) are an efficient antitumor peptide that specifically kills tumor cells. In the present study, we designed antimicrobial peptides containing the C*iso*DGRC motif (CDAK) and assessed its antitumor activity for CD13^−^/α_v_β_3_^+^ breast cancer cells (MCF-7 and MDA-MB-231) in vitro and in vivo.

**Methods:**

In vitro: We assessed the cytotoxicity of CDAK for MCF-7 and MDA-MB-231 breast cancer cells, the human umbilical vein endothelial cell (HUVEC), and human foreskin fibroblasts (HFF). We performed an apoptosis assay using Annexin-V/PI, DNA ladder, mitochondrial membrane potential, and Caspase-3 and Bcl-2. The effect on cell cycles and affinity with cell were tested using flow cytometry and fluorescent microscopy and the effect on invasion was analyzed using an invasion assay. CDAK was injected intravenously into tumor-bearing athymic nude mice in vivo experiment.

**Results:**

CDAK showed cytotoxic activity in MCF-7 and MDA-MB-231 cells, whereas HUVEC and HFF were less sensitive to the peptides. CDAK induced apoptosis, reduced mitochondrial membrane potential, promoted Caspase-3, and inhibited Bcl-2 expression in the two breast cancer cell lines. In addition, CDAK inhibited proliferation of cancer cell through S phase arrest, and own selective affinity with MCF-7 and MDA-MB-231cells, inhibited the invasion of MDA-MB-231 cells. In vivo, CDAK significant inhibited the progression of the tumor and the generation of neovascularization.

**Conclusion:**

Antimicrobial peptides containing the C*iso*DGRC (CDAK) motif could efficiently exhibit the antitumor activity for CD13^−^/α_v_β_3_^+^ breast cancer cells.

## Introduction

Various compounds and particles have been coupled or added synthetically to the Asn-Gly-Arg (NGR) motif to target recognized aminopeptidase N (CD13), which are over-expessed in angiogenic blood vessels and some tumors cells, which increases antitumor activity. This includes chemotherapeutic drugs, gene molecular, and pro-apoptosis peptides [1], [2], [3]. Some tumors cells, however, express little or do not express CD13, which reduced the efficiency of NGR. Recent research has shown that the Asn residue of NGR can deamidate and generate *iso*Asp residues to form the mixture of *iso*Asp-Gly-Arg (*iso*DGR) and Asp-Gly-Arg (DGR). The transition of NGR to *iso*DGR loses its ability to recognize CD13, but gains the property of binding to α_v_β_3_. α_v_β_3_ is a type of integrin that overexpresses on endothelial cells and tumor cells and is responsible for adhering to and migrating endothelial cells [4], [5]. Studies have shown that *iso*DGR has a more efficient affinity with α_v_β_3_ than Arg-Gly-Asp (RGD) as a well-known α_v_β_3_-binding motif [6], [7], [8], [9], [10], [11]. *iso*DGR not only has the canonical RGD/α_v_β_3_ contacts, but also establishes additional polar interactions [12]. In addition, studies have shown that the cyclic *iso*DGR (C*iso*DGRC) motif with a disulfide bridge constraint can increase the tumor targeting efficiency and stability of bent conformation [13]. C*iso*DGRC peptides as a targeted delivery of tumor necrosis factor α (TNF) has been explored and shown promising results. C*iso*DGRC thus appears to be a promising candidate as a tool for targeted delivery of anti-agents for CD13^−^/α_v_β_3_+ tumor cells, such as MCF-7 and MDA-MB-231 cell lines [14], [15].

Antimicrobial peptides (AMPs) have drawn attention as a promising alternative to current antitumor agents. Antimicrobial peptides are linear, cationic and α-helix-forming. Cationic amino acids electrostatically attract the anionic of phospholipids to disrupt negatively charged cell membranes or form non-permeabilized pore-like structures that permit translocation of the AMPs into the cytoplasm without cell lysis [16]. The outer leaflet of the cell membrane owns the negatively charged phosphatidylserine (PS) in tumor cells. The negatively charged PS, however, is localized exclusively to the inner leaflet of the membrane in normal cells [17]. The difference in the composition of the cell membrane between tumor cells and normal cells leads tumor cells to be more sensitive to AMPs. In addition, another possibility for tumor cells damage is the induction of apoptosis in cancer cells via mitochondrial membrane disruption after AMPs enter the cytoplasm [18].

In the present study, we conjugate C*iso*DGRC and AMPs to develop a novel tumor targeting AMP to exploit its antitumor properties for CD13^−^/α_v_β_3_^+^ tumor cells.

## Materials and Methods

### Design of the peptide

The sequence of antimicrobial peptides containing the C*iso*DGRC motif is shown in the following: (1) C*iso*DGRCLLIIKLAKLAKKLAKLAK (CDAK),(2) CRDGCALKAIAKLKLKKLILALK (CRLK). KLAKLAKKLAKLAK is a type of sequence from antimicrobial peptides [19]. As a linker, LLII fuses the two functional domains. CRLK was a random sequence of CDAK. Peptides were synthesized and then purified by high-performance liquid chromatography to 95% (Peptide Synthesis Facility, Guangzhou Bio Integration Biotechnology Company, Guangzhou, China), dissolved in deionized water to 1 mg/ml, and saved in −20°C.

### Cell lines

Four cell lines as follows were purchased from the cell bank of the Chinese Academy of Sciences (Shanghai, China): (1) human breast cancer cell lines MCF-7 and MDA-MB-231; (2) human foreskin fibroblasts (HFF), and (3) human umbilical vein endothelial cells (HUVEC). Cells had a monolayer long-term culture in a flask of RPMI1640 medium supplemented with 10% fetal bovine serum (FBS; Hyclone, Logan, Utah, USA).

### Cell viability assay

The MCF-7 and MDA-MB-231 cells were cultured in 96-well plates at a density of 5×10^4^ cells/well. CDAK and CRLK were added to different wells, ranging from 10 µg/ml to 200 µg/ml, respectively. After 24 h, 48 h and 72 h, 3-[4,5-dimethylthiazol- 2-yl]-2,5-diphenyltetrazolium bromide (MTT; Sigma-Aldrich, St. Louis, MO, USA) was added to each well and incubated for 4 h. The MTT was dissolved in 200 µL of Dimethyl sulfoxide (DMSO). Absorbance was detected using a microplate reader at 490 nm. Wells without peptides were used to control cell viability, representing 100% cell survival. Wells without cells were used for the blank of the spectrophotometer.

### Flow cytometry

Annexin-V/PI double notation detects apoptosis. Cells were harvested by typsinization followed by washing with phosphate buffered saline (PBS) two times after being treated with CDAK using the dose of IC50 (MCF-7 were 190 µg/ml,MDA-MB-23 were 212 µg/ml), CRLK(200 µg/ml)and control (PBS) for 24 h. They were then incubated with 500 µL Binding Buffer and Annexin-V-FITC (20 µg/ml) 10 ul at room temperature for 30 minutes. We then added propidium iodide (PI) (50 µg/ml), 5 ul for five minutes. The samples were then analyzed using flow cytometry (BD Biosciences, San Diego, CA, USA).

Mitochondrial membrane potential (Ψm) was analyzed using flow cytometry through the JC-1 mitochondrial membrane potential assay kit (Cell Technology Company, Minneapolis, MN, USA). Cells were harvested by typsinization followed by washing with PBS two times after being treated with CDAK ( the dose were 190 µg/ml and 212 µg/ml respectively in MCF-7 and MDA-MB-231), CRLK(200 µg/ml)and control (PBS) for 24 h, then incubated with 500 µL JC-1 working fluid for 20 minutes, washed with 500 µL staining solution twice, and then suspension with an incubation buffer. The cells were analyzed using flow cytometry.

The affinity rate of peptides was analyzed using flow cytometry. Cells were seeded in 6-well plates at a density of 5×10^5^ cells/well. After 24 h, the cells were treated with CDAK and CRLK (10 µg/ml; Rhodamine B was connected to the K of the amino acid residue's end) for 12 h. At the end of the incubation, the cells were harvested by trypsin and washed with PBS three times. We then proceeded with the fluorescence-activated cell sorting (FACS).

### Analysis of cell cycle dynamics and DNA fragmentation

After being incubated with CDAK (190 µg/ml and 212 µg/ml), CRLK (200 µg/ml) and control (PBS) for 24 h, 20 µM BrdU (Sigma-Aldrich, St. Louis, MO, USA) was added to the media for 30 min. Cells were harvested and fixed in iced-cold 75% ethanol for 24 h. Then, the cells were treated with 4N HCL, neutralized by 0.1 M borax, and washed with PBS containing 0.05% bovine serum albumin (BSA). The cells were incubated sequentially with anti-BrdU antibody (santa cruz biotechnology, California, US) and FITC-conjugate anti-mouse secondary antibody in the presence of 0.5%BSA and 0.5%Tween-20. Cells were added PI (50 µg/ml) and RNase A (50 µg/ml) for 20 minutes and analyzed by flow cytometry. Select a forward scatter (FSC) versus side scatter (SSC) window defined single cells and exclude cell doublets.

For DNA fragmentation analysis, DAN of cells which were treated with CDAK (190 µg/ml and 212 µg/ml), CRLK (200 µg/ml) and control (PBS) for 24 h were extracted using a selective apoptotic DNA ladder extraction KIT (Biomed Company, Beijing, China) and were analyzed using 1% agarose gels. DNA was visualized with ethidium bromide staining.

### Fluorescence microscopy detections

Cells were incubated with CDAK and CRLK (10 µg/ml; Rhodamine B was connected to the K of the amino acid residue's end) for 12 h and washed with PBS three times. They were detected by a fluorescence microscope (Ex and Em wavelengths were set at 550 nm and 620 nm, respectively, with exposure at 400 ms). Images were recorded using a CDD camera (ECLIPSE TE2000-U, Nikon, Tokyo, Japan).

### Western-blot

Cells were treated with CDAK (190 µg/ml and 212 µg/ml), CRLK (200 µg/ml) and control (PBS) for 24 h and washed with cooled PBS three times. They were then added to RIPA 140 ul and the protease inhibitors (2 mg/ml) for 30 minutes. The lysates were then centrifuged at 12,000 *g* at 4°C for 20 minutes and boiled in a loading buffer for 5 minutes. The total protein was normalized using a Bradford protein assay, and 150-microgram protein samples were loaded on sodium dodecyl sulfate-polyacrylamide gels and transferred to polyvinylidene difluoride membranes. The membrane was probed with rabbit monoclonal caspase-3, bcl-2, α_v_, β_3_, and the CD13 antibody purchased from Abcam, (Abcam, Cambridge, UK) at 4°C overnight. After washing three times in Tris hydrochloride buffer (TBS) containing 0.1% Tween-20 (TBS-T), the second antibody was incubated with the membranes for 2 h at room temperature. The membranes were then washed with TBS-T three times for 10 minutes and exposed to X-ray film. Autoradiogram signals were quantified using a gel densitometric scanning program. The relative expression of protein was determined from the optical density ratio of the corresponding protein bands.

### Invasion assay

Invasion assay was performed in a Matrigel-coated invasion chamber (Matrigel, BD Biosciences, San Diego, CA; Transwell Chamber, Millipore, Massachusetts, USA). MDA-MB-231cells were plated in the upper chamber at a density of 1×10^5^ cells/100 µl in serum-free RPMI-1640 medium and were then treated with CDAK and CRLK (10 µg/ml). The lower chamber was filled with RPMI-1640 medium containing 20% FBS. After incubating at 37°C for 24 h, the nonmigrated cells were scraped in the upper chamber with a cotton swab, and fixed the migrated cell on the lower surface of the porous membrane with methanol. The cells were then stained with crystal violet and counted by a light microscope.

### In vivo efficacy in a xenograft model

The experiment was approved by the Animal Care and Use Committee of Xi'an Jiaotong University. MDA-MB-231 cells (2×10^6^) were injected subcutaneously into the right flank of 6- to 9-week-old female BALB/cnu-nu athymic nude mice (Shanghai Silaike Laboratory Animal Co., Ltd, Shanghai, China) weighing 18 to 20 g. When the tumor reached 60 mm^3^ in size, the mice were randomized into three groups: (1) CDAK (4 mg/kg); (2) CRLK (4 mg/kg); and (3) saline (control). They were then injected intravenously (50 µL/injection) three times a week for three weeks. Tumor volume was measured three times a week using calipers to calculate the tumor size using the following formula: length×width^2^×0.5. All values are expressed as the mean ± SD.

Tumor-bearing athymic nude mice were sacrificed and the weights of the tumors were recorded. The tumor tissue, liver as well as lung tissues of mice were paraffin-embedded. The tumor paraffin sections were incubated for 10 minutes with 3% H_2_O_2_ deionized water to eliminate the endogenous peroxidase activity, washed in PBS three times for five minutes, 5% goat serum was added for 15 minutes, then incubated with mouse monoclonal CD105 antibody (Abcam, Cambridge, UK) at 4°C overnight and washed with PBS three times for five minutes. The biotin-labeled goat anti-mouse IgG were incubated with sections at 37°C for 15 minutes, and then the sections were washed by PBS three times for five minutes. Horseradish peroxidase-avidin enzyme working solution was added at 37°C for 15 minutes and washed with PBS. DAB was added to develop the color, and the nuclei were counterstained mildly with hematoxylin. The liver and lung paraffin sections were stained with hemaetoxylin and eosin (HE) and were independently evluated by two pathologists.

Terminal deoxynucleotidyl teansferase-mediated dUTP nick end-labeling (TUNEL) were examined in the lung and liver using TdT In Situ Apoptosis Detection Kit (Trevigen, Gaithersburg, Maryland, USA) following the manufacturer's protocols. Apoptosis cells were identified as having brown nuclei under a light microscope. The number of apoptosis cells was counted in five random fields (×400) in a blinded manner.

### Statistical analysis

The experiments with more than two treatment groups and various treatment concentrations were tested by univariate ANOVA, followed by Bonferroni or Dunnett's for multiple comparisons. All values are presented as the mean ± SD. An alpha level of <0.05 was used as the criterion of significance. Results were reproduced in three independent experiments.

## Results

### Test of cytotoxicity on CDAK for CD13 negative breast cancer cell

**Figure 1:**
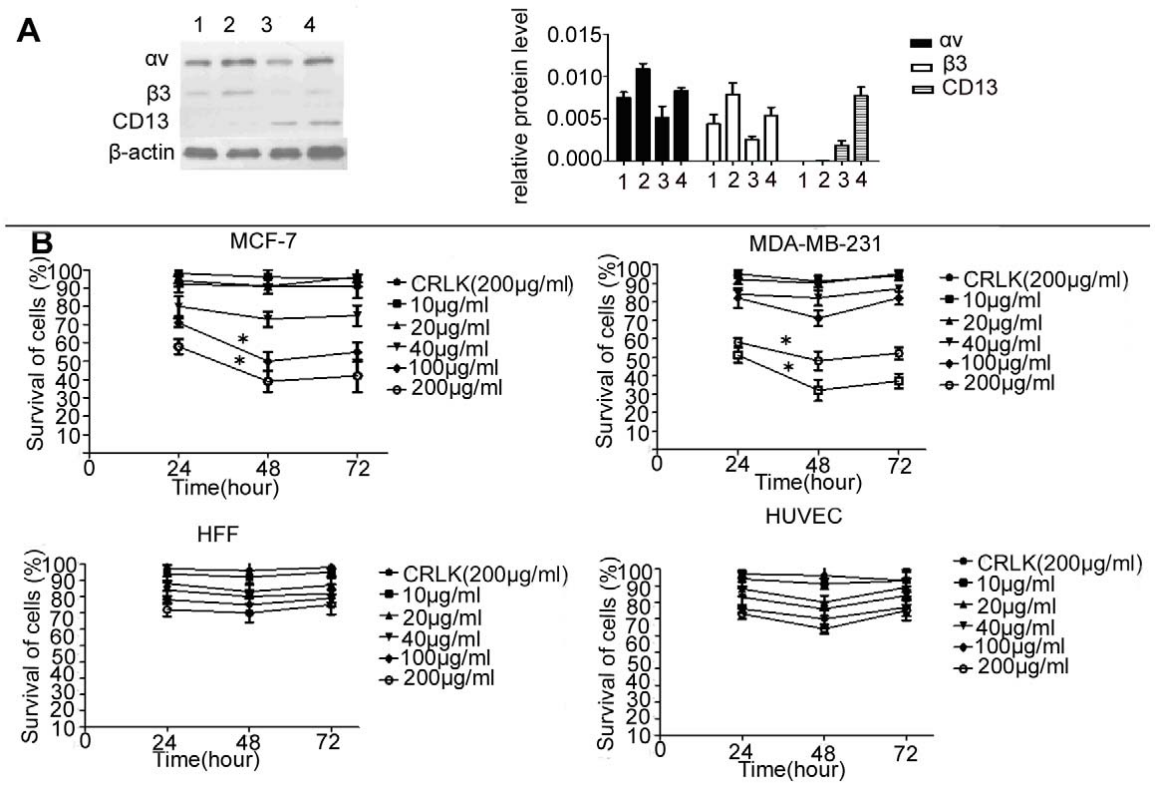
The expression status of α_v_β_3_ and CD13 and the cytotoxic activity of CDAK in cell lines. (**A**) Western-blot analyzed the protein expression of α_v_β_3_ and CD13 in the four cell lines, *Lane 1–4*, MDA-MB-231, MCF-7, HFF and HUVEC. The expression levels were analyzed by the ratio of optical density with β-actin. *P* = 0.005 (ANOVA asay) (**B**) The four cell lines were cultured with CRLK (200 µg/ml) and various concentrations of CDAK (10–200 µg/ml) for 24 h, 48 h, and 72 h. The cytotoxic activity was assessed using MTT. CDAK had significant cytotoxicity for MCF-7 and MDA-MB-231cells, *P*<0.01 (ANOVA asay). Data are presented by means ± SD (bar) from triplicate determinations. *P<0.05 versus control.

We firstly examined the expression of CD13 and α_v_β_3_ on the MCF-7, MDA-MB-231, HUVEC, and Fibroblast cells using Western-blot. As shown in Figure 1A, we did not detect the expression of CD13 in MCF-7 or MDA-MB-231cells. In contrast, the two cell lines all expressed α_v_β_3_, HUVEC and HFF cells showed a double positive expression on the protein of CD13 and α_v_β_3_. The MCF-7 and HUVEC cells expressed more α_v_β_3_ compared to MDA-MB-231 cells. CD13 was expressed higher in HUVEC than HFF. We then assessed the cytotoxic activity of CDAK on the two kinds of CD13^−^/α_v_β_3_^+^ breast cancer cells and the two normal cells lines through MTT. The results indicated that the treatment with CDAK induced dose–dependent cytotoxicity in the MCF-7 and MDA-MB-231 cells (Figure 1B). We found significant cytotoxicity at the >40 µg/ml concentration (*P*<0.05). However, the cytotoxicity decreased after 48 h. The IC50 of CDAK (the peptides' concentration that induces 50% inhibition of cell growth) were 190 µg/ml and 212 µg/ml for MCF-7 and MDA-MB-231 cells respectively at 24 h (Table 1), which were 1/4 to 1/5 compared to HUVEC and HFF cells, respectively. These data suggest that MCF-7 and MDA-MB-231 cells were susceptible to CDAK more than normal cells. There was a correlation between the expression of α_v_β and the cytotoxicity of CDAK in cancer cells.

**Table 1:** Cytotoxicity of peptides to various cell lines.

Cell line	IC_50_ (µg/ml)	P value (difference vs HFF)
MCF-7	190	0.006
MDA-MB-231	212	0.026
HUVEC	861	0.019
HFF	912	

### Characterization of the breast cancer cell death mechanism by CDAK

**Figure 2:**
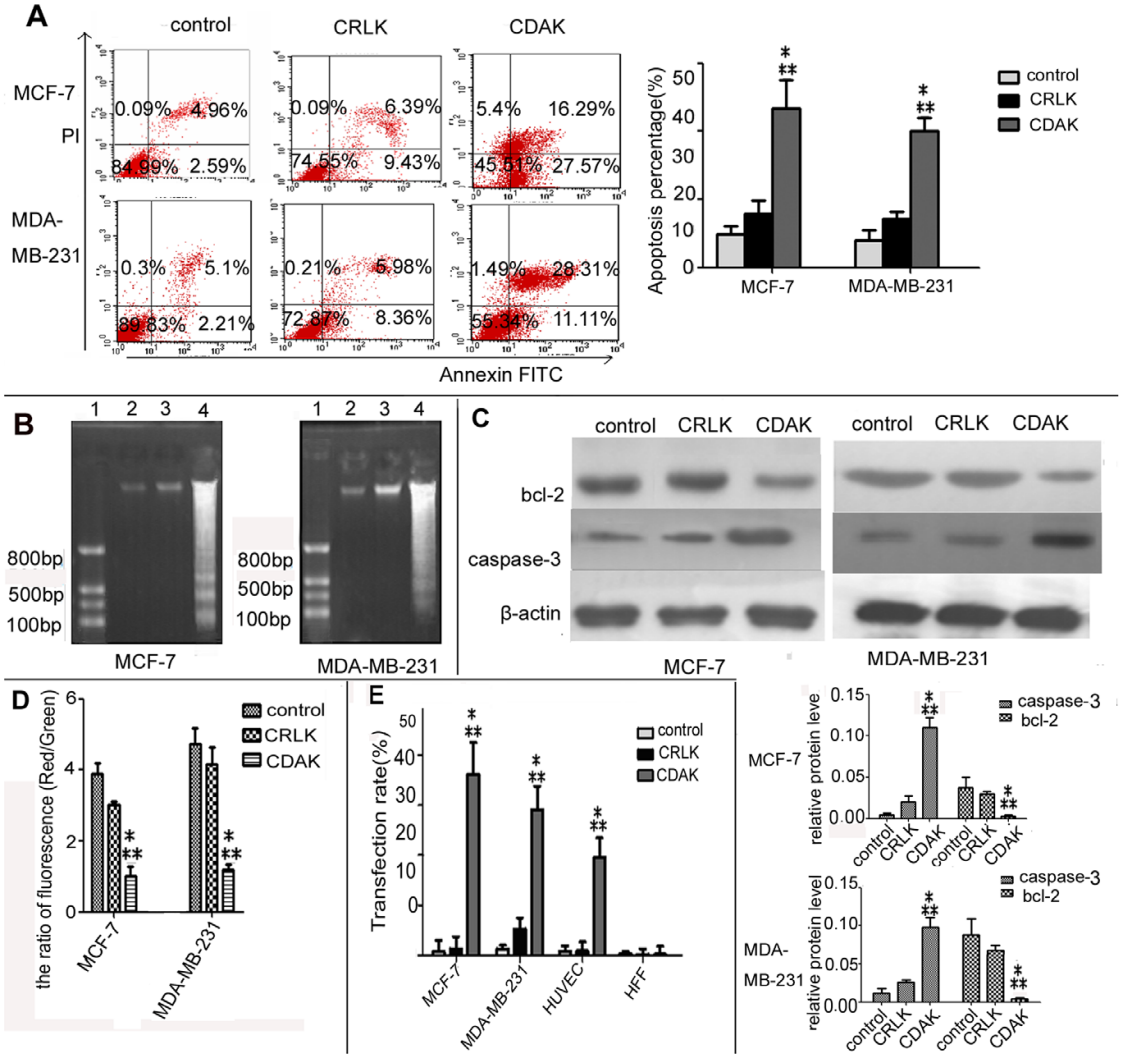
Characterization of the breast cancer cell death mechanism by CDAK. (**A**) The apoptosis of breast cancer cells treated with CRLK (200 µg/ml) and CDAK (190 µg/ml in MCF-7, 212 µg/ml in MDA-MB-231) for 24 h were analyzed using flow cytometry for Annexin V/PI stain. The sum of the upper right and lower right quadrants are expressed as a percentage of the apoptotic cell. CDAK enhanced the percentage ratio of apoptosis, *P*<0.01. (**B**) Electrophoretic analysis of DNA extracted from MCF-7 and MDA-MB-231 treated with CRLK(200 µg/ml) and CDAK(190 µg/ml,212 µg/ml) for 24 h. *Lane 1*, DNA mark; *Lane 2–4*, control, CRLK and CDAK. The CDAK group forms a clear DNA ladder. (**C**) Pro-apoptosis protein caspase-3 and inhibit protein bcl-2 were analyzed by Western-blot. The whole protein of MCF-7 and MDA-MB-231 treated with CRLK (200 µg/ml) and CDAK (190 µg/ml,212 µg/ml) for 24 h were extracted. The expression levels were analyzed by the ratio of optical density with β-actin. CDAK inhibited the expression of bcl-2 promoted caspase-3 compared with CRLK and control, *P*<0.01. (**D**) MCF-7 and MDA-MB-231 treated with CRLK (200 µg/ml) and CDAK (190 µg/ml and 212 µg/ml) for 24 h were analyzed for the mitochondrial transmembrane potential using flow cytometry and JC-1. The ratio of fluorescence (red/green) indicates the mitochondrial transmembrane potential. CDAK decreased the ration of fluorescence, *P*<0.01. (**E**) The affinity rate of CRLK and CDAK (10 µg/ml) for 12 h were analyzed using flow cytometry. MCF-7 and MDA-MB-231 showed more binding than HUVEC and HFF, especially MCF-7, *P*<0.01 (ANOVA assay). The results are represented as means± SD from triplicate determinations. * *P*<0.05 versus control; ** *P*<0.05 versus CRLK.

To explore whether apoptosis played important role in the cytotoxicity of CDAK, MCF-7 and MDA-MB-231 cells were treated with CDAK for 24 h. As shown in Figure 2A and 2B, the outcome of the Annexin V/PI detection showed that CDAK increased the percentage of apoptosis cells in both the MCF-7 and MDA-MB-231cells (*P*<0.05). The DNA ladder assay showed that no DNA ladder was detected in the MCF-7 and MDA-MB-231 cells treated with CRLK. However, the formation of DNA nucleosome ladders was clearly detected in the MCF-7 and MDA-MB-231 cells treated with CDAK. In addition, the expression of Caspase-3 and Bcl-2 were detected by Western-blot (Fig. 2C). Caspase-3 increased 8.5 times and 2.8 times in MCF-7 and MDA-MB-231cells treated with CDAK compared with control (*P*<0.05), and Bcl-2 decreased 96% and 92% in MCF-7 and MDA-MB-231cells treated with CDAK compared with control (*P*<0.05).

Previous experiments have shown that AMPs induce tumor cell apoptosis by disrupting the mitochondria. To confirm this mechanism further, mitochondria membrane potential (Ψm) was assessed using JC-1 staining. The disrupting of the mitochondria along with dropping of Ψm, and the variance of fluorescence ratio (red/green) indicate the variance of Ψm. After treated with CDAK, the mitochondria membrane potential decreased 33% and 28% respectively in MCF-7 and MDA-MB-231 cells compared to CRLK (*P*<0.05) (Figure 2D).

### Affinity of CDAK in CD13 negative breast cancer cells

**Figure 3:**
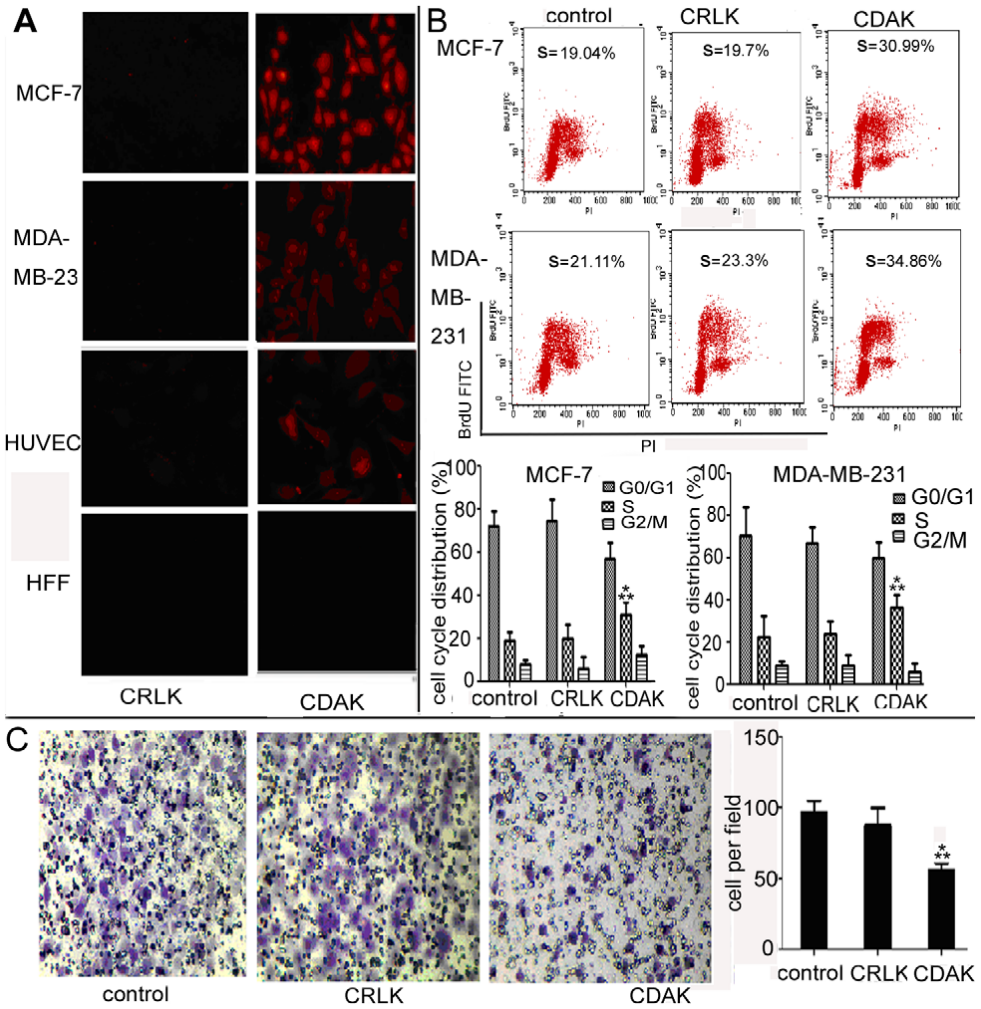
The affinity of CDAK and the effect of CDAK on cell cycle and invasion. (**A**) Fluorescence photograph of MCF-7, MDA-MB-231, HUVEC and HFF cells treated with CRLK and CDAK (10 µg/ml), which is labeled by Rhodamine B in the K of the amino acid residues' end, for 12 h (400× magnification). (**B**) MCF-7 and MDA-MB-231 cells treated with CRLK (200 µg/ml) and CDAK (190 µg/ml in MCF-7 and 212 µg/ml in MDA-MB-231) for 24 h were analyzed for the cell cycle distribution using flow cytometry for BrdU/PI stain. CDAK induced the S phage arrest, *P*<0.01 (ANOVA asay). (**C**) Representative image depicting the effect of CDAK and CRLK (10 µg/ml) on MDA-MB-231 cells invasion (400× magnification). Quantitation of migrating cells per high field, *P*<0.01 (ANOVA assay). The data are mean ± SD from triplicate determinations. * *P*<0.05 versus control ** *P*<0.05 versus CRLK.

Rhodamine B was connected to the K of the amino acid residues' end. MCF-7, MDA-MB-231, HUVEC, and Fibroblast cells were treated with CDAK and CRLK (10 µg/ml) for 12 h. The affinity rate of CDAK was then assessed by flow cytometry (Figure 2E) The result showed that CDAK had significant higher affinity rate with both MCF-7 and MDA-MB-231 cells compared to HUVEC and HFF cells (*P*<0.05). At the same time, we further assessed the affinity using fluorescence microscopy. As shown in Figure 3A, CDAK affinity was evident in the MCF-7, MDA-MB-231 and HUVEC cells, but not in the Fibroblast cells. These data further verified that C*iso*DGRC motif has the ability to specifically distinguish and bind tumor cells.

### The effect of CDAK on cell cycle in CD13 negative breast cancer cells

We analyzed the effect of CDAK on the cell cycle. The results showed that the cells were significantly arrested in the S phase after incubated with CDAK for 24 h (*P*<0.05) (Fig. 3B). The change between the control and CRLK did not show a significant difference (*P*<0.05).

### CDAK inhibits invasion of CD13 negative breast cancer cell

A transwell chamber invasion assay was performed using the MDA-MB-231 cells, which are high invasion cancer cells, to determine whether CDAK is capable of inhibiting the invasion of the MDA-MB-231cells. As shown in Figure 3C, invasion of the MDA-MB-231 cells were significantly inhibited in the CDAK compared with the control group and CRLK (*P*<0.05).

### The effect of CDAK's antitumor activity on breast cancer xenograft growth

**Figure 4:**
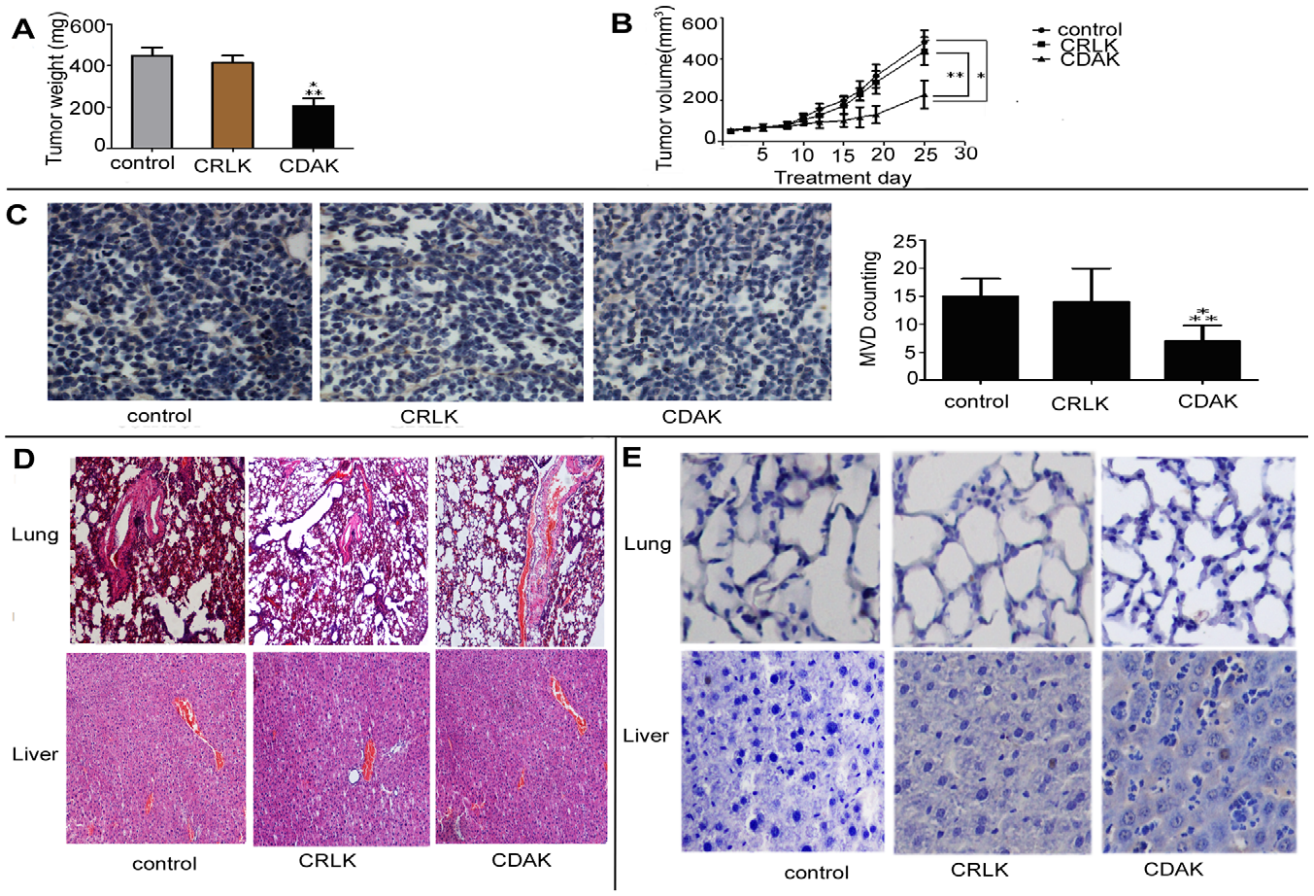
Antitumor activity of CDAK in breast cancer xenograft in vivo. (**A**) Tumor–growth curve. MDA-MB-231 cell lines were implanted into the right flank of female athymic mice. When the tumor size reached about 60 mm^3^, mice received an intravenous injection of CDAK (4 mg/kg,) and CRLK (4 mg/kg), or saline used as control (three times a week). The volumes of the tumors were measure three times a week. The tumor volume of CDAK group was smaller than control and CRLK, *P*<0.01. (**B**) The athymic mice were sacrificed as 25 d after being injected with CDAK and CRLK and the weight of each tumor was measured. The CDAK group had low weight compared to the control and the CRLK groups. *P* = 0.003. (**C**) The inhibition of angiogenesis was evaluated in tumor sections using immunohistochemistry with anti-CD105 antibody. Angiogenesis was quantified by image analysis of Microvessel density (MVD) (400× magnification). CDAK caused significant inhibition of angiogenesis. *P* = 0.003. (D) Images (200× magnification) of the lung and liver from athymic mice were obtained by staining with hemaetoxylin and eosin (HE). No significant damage was observed. (E) Representative photographs of the tumor section examined by TUNEL assay, light microscope (×400). The number of apoptotic cells was counter 5 random field in blinded manner. There differences of apoptotic cell were not statistic significant in the three groups (table 2) (ANOVA assay). The images were analyzed by pro plus 5.0. Each group had five nude mice. Data are shown as mean ± SD. *P<0.05 versus control, ** *P*<0.05 versus CRLK.

Finally, we tested activity of CDAK in female athymic nude mice implanted with MDA-MB-231 cells. The CDAK and CRLK concentration used in the present study refers to a previous animal study [20]. As shown in Figure 4A and 4B, the tumors treated with CDAK were significant smaller than the control group (*P*<0.05). The CRLK treated tumors showed a similar trend, but the differences did not reach a statistically significance compared with control group. For example, after being injected for 25 d, the average volume of tumor in control mice was about two- to three fold higher compared with mice treated with CDAK. The average weights of tumors were also lower in the CDAK group compared to the CRLK and control mice (*P*<0.05).

As shown in Figure 4C, CDAK significantly inhibited the angiogenesis while not CRLK (*P*<0.05). The data also indicated that CDAK inhibited angiongensis in vivo. No abnormalities were observed in the lung and liver of mice in the histological examination (Fig. 4D). The TUNEL assay showed that apoptosis was not significant induced by CDAK in the lung and liver compared with control (*P*>0.05) (Fig. 4E, table 2).

**Table 2:** Comparison of apoptisos ratio in liver and lung of athymic mice (mean ± SD, n = 5).

tissue	group	apoptosis rate (%)	P value (versus control)
lung	control	0.14±0.01	
	CRLK	0.17±0.01	0.64
	CDAK	0.16±0.02	0.48
liver	control	0.33%±0.01	
	CRLK	0.35%±0.23	1
	CDAK	0.38%±0.14	0.25

## Discussion

Therapy peptides are gaining increased popularity to treat cancerous tumors. This might be the case because we know that peptides are generally less expensive than antibody-based therapy due to the development of recombinant or solid-phase chemical synthesis techniques. And, compared with proteins, peptides have higher solubility in water, and lower immunogenicity [21], [22]. In the present study, we linked two functional domains to construct a novel antimicrobial peptide containing *iso*DGR motif to selectively kill CD13^−^/α_v_β_3_^+^ breast cancer cells. The results showed that the synthesized peptides have an efficient antitumor effect for CD13^−^/α_v_β_3_^+^ breast cancer cells in both vitro and vivo experiments.

CD13 were up-regulated in angiogenic blood vessels, tumor cells, pericytes, fibroblasts, and smooth muscle cells. NGR, therefore, is an efficient tool to deliver targeted treatment to these cells [23]. CD13 is not present in all tumors, in fact, it was found in only 36.2% of breast cancer patients [24]. NRG motif only indirectly killed tumor cells by disrupting the angiogenic blood vessel in CD13 negative tumor cells. Current studies, however, have discovered the *iso*DGR, a derivative of NGR, has a high affinity for α_v_β_3_, which is expressed at low levels in epithelial cells and mature endothelial cells, but is up-regulated on tumor cells and tumor endothelial cells [25]. The *iso*DGR motif is therefore a proper candidate to be used as an antitumor agent, which can be targeted to CD13^−^/α_v_β_3_^+^ tumor cells. In the present study, we connected two functional domains, antimicrobial peptides and *iso*DGR, to conjugate novel antitumor peptides that selectively bind integrin α_v_β_3_ to kill tumor cells. We used cyclic *iso*DGR, which could bind α_v_β_3_ with an affinity >100-fold higher than that of linear *iso*DGR, to target kill α_v_β_3_^+^ tumor cells. The experiment results demonstrate that the C*iso*DGRC motif has a higher affinity for MCF-7 compared to MDA-MB-231 cells, and the result of Western-blot assay shown MCF-7 have more expression of α_v_β_3_ compared to MDA-MB-231. This further verified the relationship between C*iso*DGRC motif and α_v_β_3_. Further test is needed to discriminate between normal cells and cancer cells, all of which have the expression of α_v_β_3_. The results showed that the C*iso*DGRC motif has properties to selectively recognize α_v_β_3_-positive tumor cells. Furthermore, the cytotoxicity assay demonstrated that synthesized peptides owned selective cytotoxicity for MCF-7 and MDA-MB-231cells, especially MCF-7, but left normal cells unaffected. Prior studies have shown that the antimicrobial peptides sequence used in the present study is non-toxic outside of the cell, but toxic when internalized into the cell by disrupting the mitochondrial membrane [26]. The apoptosis studies showed synthesized peptides induced the apoptosis of MCF-7 and MDA-MB-231 cells, and disrupted mitochondrial membrane potential promotes the expression of Caspase-3 and inhibits Bcl-2. At the same time, the assay of the cell cycle showed that the cells were arrested in the S phase, which demonstrates that CDAK not only promoted apoptosis, but also inhibited the proliferation of MCF-7 and MDA-MB-231cells. In the vivo experiment, the synthesized peptides significantly inhibited the xenograft growth. Other studies have shown that some antitumor agents adding the ligand of α_v_β_3_, such as RGD [27], which could inhibit tumor angiogenesis that have a high expression of α_v_β_3_. We used CD105 to stain tumor angiogenesis, and the result verified that CDAK significantly inhibited tumor angiogenesis. Regarding how the mechanism inhibited angiogenesis, α_v_β_3_ is expressed on endothelial cell during neovascularization, but is not strongly expressed on quiescent endothelial cell [28]. We speculated, therefore, that AMPs containing C*iso*DGRC could target recognized endothelial cell of angiogenesis to induce the apoptosis. We will verify this in our next experiment. At the same time, the histochemical and TUNEL assays showed the peptides had little effect on normal tissue.

AMPs containing C*iso*DGRC, like all targeting antitumor drugs, offers the possibility of targeting the CD13^−^/α_v_β_3_^+^ breast cancer cells. The results of the present experiment showed that the synthesized AMPs owned a high selectivity for CD13^−^/α_v_β_3_^+^ breast cancer cell and less cytotoxity toward normal cells. The selective property provides the efficient antitumor activity. Further studies to examine the mechanisms of antitumors, safety, immunogenicity, and antitumor spectrum will broaden the indication of AMPs containing C*iso*DGRC for the future.

## Conclusion

The present study found that AMPs containing C*iso*DGRC selectively kill CD13^−^/α_v_β_3_^+^ breast cancer cells by inducing apoptosis and inhibiting proliferation. Furthermore, the vivo experiments showed that AMPs containing C*iso*DGRC could significantly inhibited CD13^−^/α_v_β_3_^+^ tumor progression and angiogenesis.
